# Polydeoxyribonucleotide: A Promising Biological Platform to Accelerate Impaired Skin Wound Healing

**DOI:** 10.3390/ph14111103

**Published:** 2021-10-29

**Authors:** Mariarosaria Galeano, Giovanni Pallio, Natasha Irrera, Federica Mannino, Alessandra Bitto, Domenica Altavilla, Mario Vaccaro, Giovanni Squadrito, Vincenzo Arcoraci, Michele Rosario Colonna, Rita Lauro, Francesco Squadrito

**Affiliations:** 1 Department of Human Pathology and Evolutive Age “Gaetano Barresi”, University of Messina, Via C. Valeria, 98125 Messina, Italy; mariarosaria.galeano@unime.it (M.G.); michelerosario.colonna@unime.it (M.R.C.); 2 Department of Clinical and Experimental Medicine, University of Messina, Via C. Valeria, 98125 Messina, Italy; gpallio@unime.it (G.P.); nirrera@unime.it (N.I.); fmannino@unime.it (F.M.); abitto@unime.it (A.B.); mario.vaccaro@unime.it (M.V.); giovanni.squadrito@unime.it (G.S.); varcoraci@unime.it (V.A.); rita.lauro@studenti.unime.it (R.L.); 3 SunNutraPharma, Academic Spin-Off Company of the University of Messina, Via C. Valeria, 98125 Messina, Italy; daltavilla@unime.it; 4 Department of Biomedical and Dental Sciences and Morphological and Functional Imaging, University of Messina, Via C. Valeria, 98125 Messina, Italy

**Keywords:** polydeoxyribonucleotide, PDRN, adenosine receptors, wound healing

## Abstract

The normal wound healing process is characterized by a complex, highly integrated cascade of events, requiring the interactions of many cell types, including inflammatory cells, fibroblasts, keratinocytes and endothelial cells, as well as the involvement of growth factors and enzymes. However, several diseases such as diabetes, thermal injury and ischemia could lead to an impaired wound healing process characterized by wound hypoxia, high levels of oxygen radicals, reduced angiogenesis, decreased collagen synthesis and organization. Polydeoxyribonucleotide (PDRN) has been used to improve wound healing through local and systemic administration thanks to its ability to promote cell migration and growth, angiogenesis, and to reduce inflammation on impaired wound healing models in vitro, in vivo and clinical studies. In light of all these observations, the aim of this review is to provide a full overview of PDRN applications on skin regeneration. We reviewed papers published in the last 25 years on PubMed, inserting “polydeoxyribonucleotide and wound healing” as the main search term. All data obtained proved the ability of PDRN in promoting physiological tissue repair through adenosine A_2A_ receptor activation and salvage pathway suggesting that PDRN has proven encouraging results in terms of healing time, wound regeneration and absence of side effects.

## 1. Introduction

The skin, the outermost part of the body, provides a natural barrier against the environment and exerts a variety of essential protective functions. Upon disruption of skin integrity, either from active injuries or chronic insults, a multi-step process is initiated, leading to the reconstruction of wounded tissue and reestablishment of skin barrier functions. In physiological conditions it is established a normal wound healing process characterized by a complex and highly integrated cascade of events, requiring the interactions of many cell types, including inflammatory cells, fibroblasts, keratinocytes and endothelial cells, as well as the involvement of growth factors and enzymes. Normal wound healing proceeds through four overlapping but distinct phases: hemostasis, inflammation, proliferation and maturation or remodeling [1,2]. On the other hand, several diseases such as diabetes, thermal injury and ischemia could lead to an impaired wound healing process characterized by wound hypoxia, high levels of oxygen radicals, elevated levels of matrix metalloproteases, delayed cellular infiltration and granulation tissue formation, reduced angiogenesis, decreased collagen synthesis and organization [3,4].

Impaired wound healing is a major clinical problem in patients with diabetes and is the leading cause of lower extremity amputation in the so-called “diabetic foot disease” [5,6]. Diabetic foot ulcers readily become chronic, and chronic ulcers have biological properties that differ substantially from acute ones. The exact mechanism by which diabetes impairs healing is not fully understood but may include abnormalities in inflammatory cell response, impaired neovascularization, decreased synthesis of collagen and increased levels of proteinases [7,8]. At the cellular level, Ferguson and colleagues demonstrated an increase in acute inflammatory cells, absence of cellular growth and migration of the epidermis over the wound, together with narrowing or occlusion of the blood vessels within the edge of the wound [9]. Patients with diabetes have impaired leukocyte function and the metabolic abnormalities of diabetes lead to dysregulated migration of neutrophils and macrophages to the wound together with reduced chemotaxis of leukocytes and an increased risk of wound infection [7].

Burn injury is another disease associated with impaired wound healing [10]. In fact, dysregulation and dysfunctional activation of the immune/inflammatory systems (in particular of the polymorphonuclear granulocytes) is a significant hallmark of the response to burn injury [11]. Thus, immediately after thermal injury, polymorphonuclear neutrophil leukocytes (PMNs) invade the lesion, provoking the release of large amounts of oxygen free radicals and proteases that will cause endothelial cell and skin damage [12]. Oxygen-free radicals participate in a number of pathophysiological processes related to burn wounds such as increasing the capillary permeability and promoting the formation of edema in the zone of stasis [13,14]. The overproduction of free radicals and proteases impairs the availability of nitric oxide (NO) and the production and release of growth factors, such as the powerful angiogenic factor VEGF. NO also possesses several biological roles in the process of cutaneous wound healing including regulation of vasodilatation, antimicrobial activity, cell proliferation, VEGF-induced neoangiogenesis, matrix deposition and remodeling. Moreover, NO also plays an important role in the regulation of burn edema and in maintaining adequate perfusion in the burn area. On the other hand, NO can present opposite biological effects depending on the presence or absence of reactive oxygen species. NO interacts with superoxide anions to form peroxynitrite responsible for decreased NO bioavailability, and as a consequence, delayed wound healing [15,16,17].

**Figure 1:**
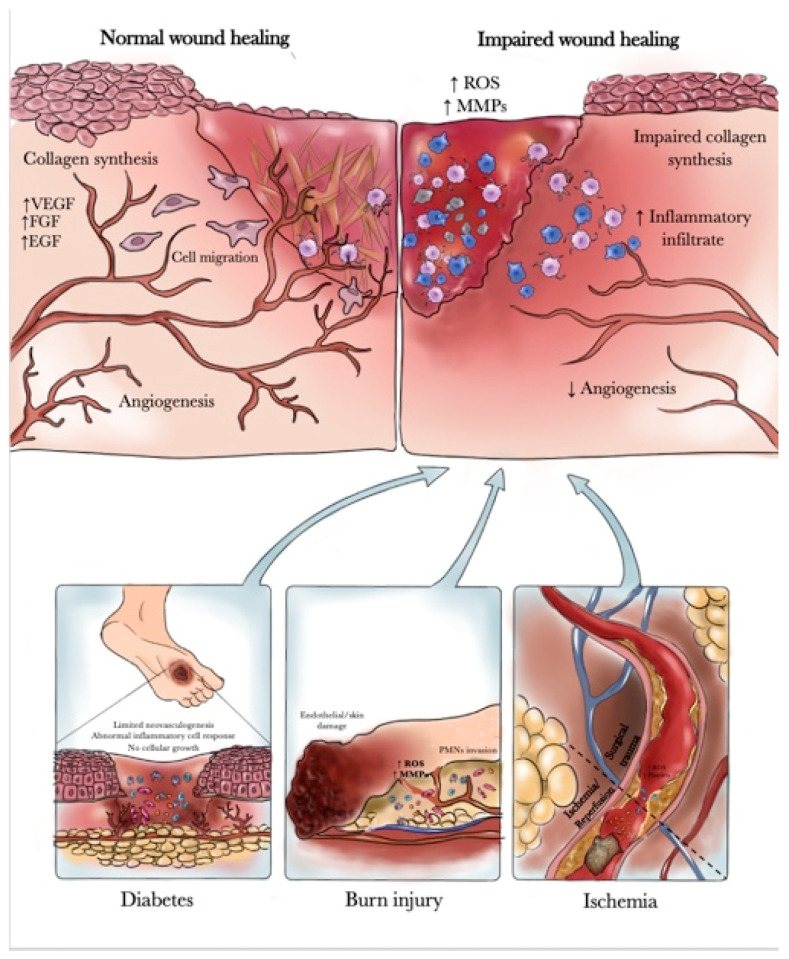
Molecular mechanism in Impaired wound healing.

Another disease that could cause impairment of wound healing is ischemia. Indeed, in reconstructive flap surgery, ischemic tissue damage may not only jeopardize wound healing but also compromise flap survival confirming that ischemic tissue necrosis remains a leading cause of flap failure that frequently occurs in the portion of the flap lying most distally to the pedicle [18]. Wound healing in ischemic tissues, such as distal flap margins, is still a source of considerable morbidity in surgical practice due to the inadequate blood supply. Consequently, local hypoxia, which decreases granulation tissue formation, collagen production, and fibroblast replication, is the key factor that strongly impairs epithelialization and biomechanical strength parameters in ischemic wounds [19] (Figure 1). Presently, various approaches to healing wounds are available such as biologics, biomaterials, stem cells therapies and skin bioprinting [20]. All these options could be attractive but several limitations in their use are present, in particular, the use of biologicals is limited due to possible contraindications, side effects and remarkable costs [20]. Furthermore, biomaterials that are an important part of modern wound care to accelerate the healing process despite the wide range of products already available have no or only a minimal effect on the healing of complex wounds [20]. Moreover, stem cell therapy represents a promising new approach to wound healing, with the potential to regenerate tissue to its pre-injured state, but additional basic and clinical studies are needed before stem cell therapy can become a treatment option for wound healing [21]. Additionally, skin bioprinting showed promise in engineering skin even if the research is still in its infancy. In particular, vascularity, optimal cell and scaffold combinations, and cost of bioprinted skin are hurdles that need to be overcome before the clinical applicability can be realized [22]. In this scenario, PDRN a compound obtained from sperm trout through an extraction process has been widely used to improve wound healing through local and systemic administration thanks to its ability to promote cell migration and growth, angiogenesis, and to reduce inflammation on impaired wound healing models including in vitro, in vivo and clinical studies. PDRN is currently on the market, it is well tolerated and has shown a very good safety profile in several clinical trials [23]. In light of all these observations, the aim of this review is to provide a full overview of PDRN applications on skin regeneration.

### Polydeoxyribonucleotide

Polydeoxyribonucleotide (PDRN) is a DNA-derived drug from *Oncorhynchus mykiss* (Salmon trout) or *Oncorhynchus keta* (Chum Salmon) sperm with molecular weights between 50 and 1500 KDa and a chain length ranging from 50 bp to 2000 bp. The drug is obtained through an extractive process at a high temperature in order to purify the substance and ensure a very high percentage of DNA. The final product is a >95% pure active principle without pharmacologically active proteins and peptides (Registration Dossier, Italian Ministry of Health) [24]. The chemical structure of PDRN consists of a low molecular weight fraction of DNA, composed of a linear polymer of deoxyribonucleotides with phosphodiester bonds in which the monomer units are represented by purine and pyrimidine nucleotides. These polymeric chains are coupled to form a steric structure defined as a double helix. The monomer unit of the PDRN chain is the deoxyribonucleotide and it is made up of three components: sugar pentose, phosphoric acid, and a purine or pyrimidine base that is connected to the pentose in position 1 through a beta-N-glycoside bond. It is likely that PDRN is cleaved by active cell membrane enzymes, providing a source for purine and pyrimidine of different tissues. Moreover, nucleotides and nucleosides have shown a synergic effect with different growth factors and can influence their production [25,26]. Furthermore, PDRN has been shown to enhance the growth rate of numerous cells such as fibroblasts, chondrocytes, preadipocytes and osteoblasts in primary cultures at therapeutic concentrations [27,28,29,30,31,32]. All these favorable effects on cell proliferation enhancement appear to be mediated by the salvage metabolic pathways and by the activation of adenosine receptors A_2a_ that has been shown to accelerate the resolution of acute inflammation, upregulate VEGF promoting vessel formation by endothelial cells and support granulation tissue formation [33].

## 2. Methodology

The database used to retrieve the papers was PubMed and the search terms used were: Polydeoxyribonucleotide and wound healing”, “Polydeoxyribonucleotide and impaired wound healing”, “Polydeoxyribonucleotide and diabetes”, “Polydeoxyribonucleotide and thermal injury”, “Polydeoxyribonucleotide and ischemia”. Papers published in the last 25 years (1996–2021) were included in order to provide a widespread overview of PDRN effectiveness in impaired wound healing.

## 3. Results

The available evidence on the role of purinergic agonists on wound healing has provided a rationale to investigate the regenerative effects of PDRN. Most experimental studies focusing on the PDRN effects have confirmed its potential in tissue regeneration in different pathologies and with different dosage and administration routes, from topical application to injection. For clarity, we have organized these studies according to their experimental model.

### 3.1. In Vitro Studies

Most in vitro studies on wound healing have been focused on fibroblasts. In this context, Thellung et al. investigated the mechanism involved in fibroblast proliferation after PDRN stimulation. PDRN was compared to adenosine in primary cultures of human skin fibroblasts and it was seen that both compounds induced cell growth. The effects of PDRN were abolished by concomitant incubation with an adenosine A_2a_ receptor antagonist, 3,7-Dimethyl-1-propargylxanthine (DMPX). It has been hypothesized that PDRN acts preferentially on the adenosine A_2a_ receptor thus suggesting the involvement of this receptor subtype in PDRN effects [28]. In further studies, cultured fibroblasts were incubated with radioactive amino acids in the presence of PDRN. Cell growth was accompanied by the internalization of PDRN-derived nucleotides utilized for the salvage pathway. In fact, damaged or hypoxic tissue often cannot undergo the DNA de novo synthesis. The salvaged bases can be transformed into nucleotides and reincorporated into DNA. PDRN generates nucleotides and nucleosides that can contribute to DNA formation, reactivating normal cell proliferation and growth pattern [34].

PDRN may also protect cells from UV-induced DNA damage. Exposure of cultured human dermal fibroblasts to ultraviolet B radiation causes the accumulation of dangerous photoproducts such as cyclobutane pyrimidine dimers (CPDS). PDRN addition to cell culture immediately after irradiation results in reduced levels of CPDS, in p53 protein activation and in enhancing DNA repair likely due to the triggering of the salvage pathway [35]. Moreover, PDRN enhances the growth of human corneal fibroblasts in primary culture depending upon the donor’s age. In particular, this effect is very consistently reproducible with donors over 60 years of age, suggesting a selective benefit of PDRN in the tissue culture of senescent cells [36].

Another important aspect in regenerative medicine is the PDRN’s ability to promote in vitro the proliferation of human pre-adipocytes, confirming that PDRN may be used for therapeutic and regenerative purposes [31].

### 3.2. Animal Studies

Skin wound healing is among the most studied applications of PDRN. PDRN effectiveness has been investigated in an incisional model of diabetes-impaired wound healing. The healing-promoting effect was supported by a marked increase in wound-breaking strength, and in the expression of VEGF, a regulator of angiogenesis that is impaired in diabetes-related wound disorders. Angiogenesis improvement was also confirmed by an increase in CD 31, transglutaminase-II, and angiopoietin, factors contributing to new vessel formation. These effects were abrogated by DMPX, thus suggesting the involvement of the adenosine A_2a_ receptor [37]. Similar outcomes were also observed in an excisional wound model of diabetic mice [38]. The wound healing properties of PDRN might be the consequence of the stimulation of the altered cell-cycle machinery that is deeply impaired in several conditions, such as diabetes [39]. Diabetic animals showed a reduced expression of the cyclin D1/CDK6 and cyclin E/CDK2 complexes at day 6 after wounding when compared with nondiabetic animals. By contrast, diabetic mice at day 6 post-wounding showed an enhanced expression of either p15 or p27, indicating that the over-expression of these negative regulators might keep granulation tissue from proliferating. Interestingly, during wound repair in genetically diabetic mice, PDRN stimulated the proliferation of granulation tissue by activating cyclin-driven cell-cycle progression (cyclin D1/CDK6, cyclin E/CDK2) and turning off the cell-cycle negative regulators p15 and p27. Adenosine A_2a_ receptor activation also results in a potent anti-inflammatory effect that can be correlated to decreasing scar formation. In this respect, Jeong et al. investigated scar prevention and wound healing in a rat incisional wound-healing model after injection of PDRN. In the PDRN group histological and immunohistochemical analyses showed reduced inflammatory cell infiltrate, more collagen deposition, and complete wound re-epithelialization, while western blot analysis revealed increased levels of type I and type III collagen [40].

Shin et al. in a wound model on diabetic mice used a PDRN-loaded Alg hydrogel that had the advantage of allowing the continuous release of the drug and eliminating the use of needles. The Alg-PDRN-treated group showed the quickest healing rate compared to other groups (saline, PDRN injection, Alg hydrogel) with a significantly higher collagen density, a reduced number of inflammatory cells and an increased expression of VEGF and α-SMA [41].

Another clinical situation characterized by a poor skin repair process and impaired angiogenesis is thermal injury. PDRN effects were investigated in mice with a deep-dermal second-degree burn injury. Systemic administration of this drug enhanced burn wound re-epithelialization and decreased time to final wound closure. PDRN improved healing of burn wounds by reducing inflammatory infiltration and burn edema, and by stimulating dermal and epidermal regeneration, proliferation of fibroblasts, and neoangiogenesis as suggested by the marked increase in microvessel density and the robust expression of PECAM-1 in the skin samples. PDRN also showed a marked systemic effect by reducing the serum levels of the pleiotropic cytokine tumor necrosis factor (TNF-α) and by augmenting wound VEGF, eNOS, iNOS expression and the wound content of nitric oxide products [42].

Among local skin flaps, random pattern skin flaps are commonly used in plastic and reconstructive surgery for skin defect repairs. The main drawback of such flaps is partial or complete distal flap necrosis. Therefore, several therapeutic approaches have been tried in many experimental models to enhance skin vitality, although the results have been controversial. In an experimental ischemic skin flap model (H-shaped double-flap), PDRN improved wound healing with complete re-epithelialization, and blood flow, evaluated by laser Doppler, by enhancing VEGF and iNOS expression and reducing HIF-1α [43]. These results have been further confirmed by Chung et al. and Lee et al. [44,45], suggesting a role for this drug in improving skin flap survival.

### 3.3. Clinical Studies

Many clinical studies have shown the positive effects of PDRN when used in plastic and reconstructive surgery. In a first double-blind, randomized, placebo-controlled study the effects of intramuscular and subcutaneous PDRN in promoting wound healing in skin explants were evaluated. A statistically significant difference in the percentage of re-epithelialization was observed in a PDRN group compared to a placebo group [46]. PDRN was later tested locally in the healing of autologous skin graft donor sites. The patients were randomized into two groups: the control group received dressing with gauzes embedded in chloramine solution; the study group received the same treatment plus PDRN ointment. PDRN dressing improved re-epithelialization and the time to complete wound healing [47].

**Figure 2:**
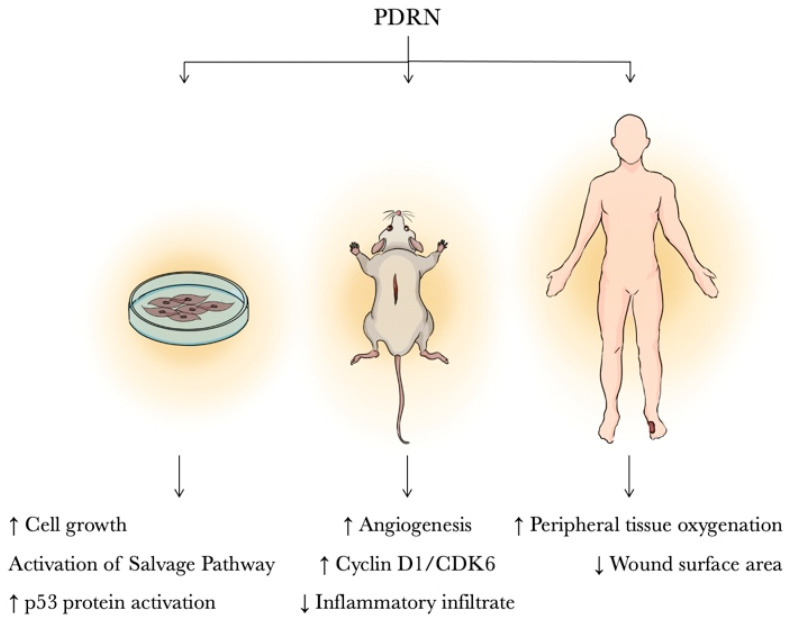
PDRN effects in: in vitro, animal and clinical studies.

Moreover, in a large double-blind randomized controlled trial (RCT) study 216 diabetic patients with Wagner grade 1 or 2 ulcers were assigned to receive placebo (number 106) or PDRN (number 110) for 8 weeks. The drug was injected daily by intramuscular route for 5 days/week and by perilesional route 2 days/week for 8 weeks. The treated group doubled the rate of complete healing of difficult-to-heal foot ulcers compared to the placebo as early as 8 weeks after the start of treatment [48]. Successively, Kim et al. performed a randomized controlled clinical study with fewer patients (n = 20) suffering from Wagner grade 1 to 4 diabetic foot ulcers. After initial surgical debridement, 10 patients were administered with a saline solution while the other 10 patients were injected with PDRN both via intramuscular and perilesional routes for two weeks. PDRN improved peripheral tissue oxygenation, increased angiogenesis and reduced inflammatory infiltrate, compared to the control group [49]. The efficacy of PDRN on pressure ulcers has also been studied; in a randomized clinical trial, the effects of PDRN were compared between a control group and a treated group. The latter was administered PDRN intramuscularly for two weeks and perilesional for 4 weeks. At the end of the treatment period, it was seen that PDRN administration reduced the wound surface area and the PUSH score, without adverse effect during treatment [50] (Table 1 and Figure 2).

## 4. Discussion

PDRN is a prodrug capable of promoting wound healing impaired by diverse pathological conditions such as diabetes, burns and ischemia. Preclinical and clinical studies have laid the basis to understand the PDRN mechanism of action. The positive effects observed are a direct consequence of the activation of adenosine A_2a_ receptors and the salvage pathway.

Adenosine is a ubiquitous purine nucleoside that can form intracellularly from ATP, ADP or AMP or extracellularly from ATP or ADP. Adenosine modulates cellular and organ function via interaction with four specific P1 G-protein associated cell surface receptors (A_1_, A_2a_, A_2b_, and A_3_) which modulate adenylate cyclase activity in an inhibitory (A_1_, A_3_) or stimulatory (A_2a_, A_2b_) fashion [51,52]. The binding of PDRN derivates to A_2a_ receptors stimulates an increase in intracellular cyclic adenosine monophosphate (cAMP) with consequent activation of the protein kinase A (PKA), which plays a key role in cell growth, survival, death, and differentiation. Adenosine A_2a_ receptors, in particular, are expressed on most cell types involved in wound healing, including macrophages, fibroblasts and microvascular endothelial cells [53,54,55]. It has been shown that activation of the adenosine receptors A_2a_ reduces inflammatory infiltration, promotes endothelial cell proliferation and migration, and VEGF production, stimulates fibroblast differentiation and maturation, consequently accelerating the repair process [56,57]. The several effects of PDRN are blocked by an adenosine A_2_ receptor antagonist, 3,7-Dimethyl-1-propargylxanthine (DMPX), which has more affinity for A_2a_ than for A_2b_ receptor subtypes [28,32].

Moreover, A_2a_ receptor activation by PDRN influences cyclins, but the molecular mechanism is still far from being fully understood. It could be hypothesized that the increase in cellular cAMP induced by receptor triggering might be responsible for this effect. Alternatively, other intracellular transduction signals such as PKA activation or phosphorylation of downstream proteins may play a significant role. The selectivity of PDRN for only the A_2a_ purinergic receptor might be due to its resistance to 5′-esonucleclease degradation that would lead to the production of small-size fragments that can bind the several types of purinergic receptors. This selectivity for the A_2a_ receptor might also explain the reason why PDRN does not produce the same collateral effects as adenosine or A_2a_ agonists which, in contrast, undergo 5′-esonucleclease degradation. Interestingly, PDRN-induced angiogenesis was not observed in other important organs such as the brain, liver, skeletal muscle, and heart.

**Figure 3:**
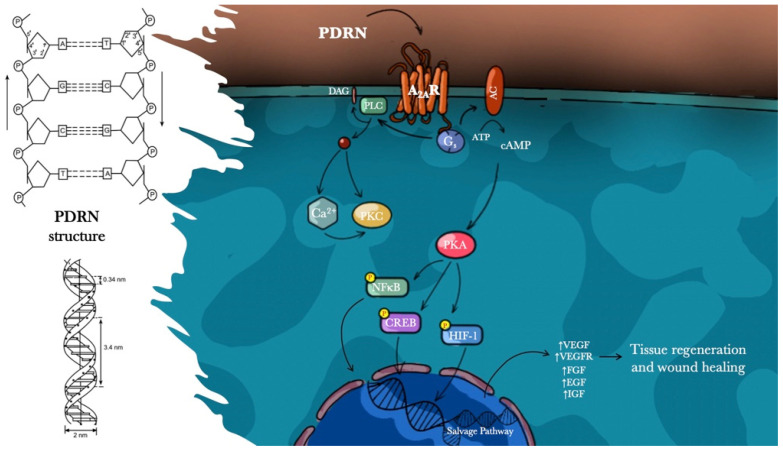
Influence of PDRN on wound healing.

Furthermore, another action mechanism, known as the “salvage pathway”, has been suggested. PDRN generates nucleotides and nucleosides that can act by promoting DNA synthesis or repairing and restoring cell proliferation and growth. This efficient energy-saving metabolic pathway represents a unique feature not shared with other DNA-derived drugs from different origins, molecular weights and manufacturing processes (Figure 3). 

Regarding safety, acute and chronic toxicity studies in mice and rats were undertaken to evaluate the effects of repeated systemic administration of PDRN, which showed no toxic effect in the brain, liver, lungs, skeletal muscle and heart, and did not cause mortality [37]. In a clinical study on the effects of PDRN on the healing of chronic diabetic foot ulcers, safety and tolerability were excellent [48].

Further studies are necessary to investigate alternative PDRN administration routes that have achieved promising results in experimental models of wound healing [41]. Specifically, the combination with bioactive materials including hydrogels, scaffolds, nanofibers and films might enhance the pharmacological activity of PDRN and offer important advantages in wound dressing and skin tissue engineering.

## Figures and Tables

**Table 1:** The table summarizes the in vitro, in vivo and clinical studies investigating the effects of polydeoxyribonucleotide on wound healing.

Study	Experimental Model	PDRN DOSES	PDRN Effects	References
Thellung et al., 1999	In vitro study Human Skin fibroblasts	10 mg/mL	↑ Cell growth	[28]
Raposio et al., 2008	In vitro study Human Pre-adipocytes	80–100 mg/mL	↑ Cell proliferation	[31]
Sini et al., 1999	In vitro study Human Skin fibroblasts	80 mg/mL	↑ Cell growth	[34]
Belletti et al., 2007	In vitro study Human skin fibroblasts	100 mg/mL	↑ DNA repair	[35]
Muratori et al., 2003	In vitro study Human Corneal fibroblasts	100 mg/mL	↑ Cell growth	[36]
Galeano et al., 2008	Animal study C57BL/ksJ-m+/+Lept^db^ (db+/db+) and (db+/+m)	8 mg/kg	↑ Wound-breaking strength ↑ VEGF expression	[37]
Kwon et al., 2019	Animal study BKS.Cg-+Lepr^db^/+Lepr^db^ and ^m^+/Lepr^db^	0.09 mg/mice	↑ Wound-breaking strength ↑ VEGF expression	[38]
Altavilla et al., 2011	Animal study C57BL/ksJ-m+/+Lept^db^ (db+/db+) and (db+/+m)	8 mg/kg	↑ Cyclin D1/CDK6, ↑ Cyclin E/CDK2 ↓ p15 and p27	[39]
Jeong et al., 2017	Animal study Sprague-Dawley	8 mg/kg	↓ Inflammatory cell infiltrate ↑ Collagen deposition	[40]
Shin et al., 2020	Animal study C57BLKS/J -db/db	100 mg/mL	↑ Collagen density ↑ VEGF and α-SMA ↓ Number of inflammatory cells	[41]
Bitto et al., 2008	Animal study C57BL/6	8 mg/kg	↓ TNF-α wound ↑ VEGF, eNOS, iNOS	[42]
Polito et al., 2012	Animal study Sprague-Dawley	8 mg/kg	↑ VEGF and iNOS ↓ HIF-1α	[43]
Chung et al., 2013	Animal study Sprague-Dawley	8 mg/kg	↑ VEGF, PECAM, CD31	[44]
Lee et al., 2015	Animal study Sprague-Dawley	8 mg/kg	↑ VEGF and CD31	[45]
Rubegni et al., 2001	Clinical study	5.625 mg/die	↑ Re-epithelialisation	[46]
Valdatta et al., 2004	Clinical study	5.625 mg/die	↑ Re-epithelialisation ↓ Time to complete wound healing	[47]
Squadrito et al., 2014	Clinical study	5.625 mg/die	↑ Re-epithelialisation ↓ Time to complete wound healing	[48]
Kim et al., 2017	Clinical study	5.625 mg/die	↑ Tissue oxygenation ↓ Inflammatory infiltrate	[49]
Kim et al., 2014	Clinical study	5.625 mg/die	↓ Wound size ↓ PUSH score	[50]

## References

[1] Singer A.J., Clark R.A.F. (1999). Cutaneous wound healing. N. Engl. J. Med..

[2] Gurtner G.C., Werner S., Barrandon Y., Longaker M.T. (2008). Wound repair and regeneration. Nature.

[3] Menke N.B., Ward K.R., Witten T.M., Bonchev D.G., Diegelmann R.F. (2007). Impaired wound healing. Clin. Dermatol..

[4] Greenhalgh D.G., Sprugel K.H., Murray M.J., Ross R. (1990). PDGF and FGF stimulate wound healing in the genetically diabetic mouse. Am. J. Pathol..

[5] Falanga V. (2005). Wound healing and its impairment in the diabetic foot. Lancet.

[6] Blakytny R., Jude E. (2006). The molecular biology of chronic wounds and delayed healing in diabetes. Diabet. Med..

[7] Delamaire M., Maugendre D., Moreno M., Le Goff M.C., Allannic H., Genetet B. (1997). Impaired leucocyte functions in diabetic patients. Diabet. Med..

[8] Altavilla D., Saitta A., Cucinotta D., Galeano M., Deodato B., Colonna M., Torre V., Russo G., Sardella A., Urna G. (2001). Inhibition of lipid peroxidation restores impaired vascular endothelial growth factor expression and stimulates wound healing and angiogenesis in the genetically diabetic mouse. Diabetes.

[9] Ferguson M.W., Herrick S.E., Spencer M.J., Shaw J.E., Boulton A.J., Sloan P. (1996). The histology of diabetic foot ulcers. Diabet. Med..

[10] Ipaktchi K., Vogt P.M. (2009). Immunology and sepsis syndrome in burn trauma. Unfallchirurg.

[11] Evers L.H., Bhavsar D., Mailänder P. (2010). The biology of burn injury. Exp. Dermatol..

[12] Arturson G. (2000). Forty years in burns research—The postburn inflammatory response. Burns.

[13] Rawlingson A. (2003). Nitric oxide, inflammation and acute burn injury. Burns.

[14] Lund T., Onarheim H., Reed R.K. (1992). Pathogenesis of edema formation in burn injuries. World J. Surg..

[15] Shi H.P., Most D., Efron D.T., Tantry U., Fischel M.H., Barbul A. (2001). The role of iNOS in wound healing. Surgery.

[16] Witte M.B., Barbul A. (2002). Role of nitric oxide in wound repair. Am. J. Surg..

[17] Lindblom L., Cassuto J., Yregård L., Mattsson U., Tarnow P., Sinclair R. (2000). Importance of nitric oxide in the regulation of burn oedema, proteinuria and urine output. Burns.

[18] Sloan G.M., Reinsch J.F. (1985). Flap physiology and the prediction of flap viability. Hand Clin..

[19] Khomullo G.V., Lotova V.I., Cherniaev A.N., Vinogradov I.N. (1986). Effect of hypoxia on DNA synthesis and collagen levels in regenerating skin. Kosm. Biol. Aviakosm. Med..

[20] Kathawala M.H., Ng W.L., Liu D., Naing M.W., Yeong W.Y., Spiller K.L., Van Dyke M., Ng K.W. (2019). Healing of Chronic Wounds: An Update of Recent Developments and Future Possibilities. Tissue Eng. Part B Rev..

[21] Kosaric N., Kiwanuka H., Gurtner G.C. (2019). Stem cell therapies for wound healing. Expert Opin. Biol. Ther..

[22] Ng W.L., Wang S., Yeong W.Y., Naing M.W. (2016). Skin Bioprinting: Impending Reality or Fantasy?. Trends Biotechnol..

[23] Kim M.S., Cho R.K., In Y. (2019). The efficacy and safety of polydeoxyribonucleotide for the treatment of knee osteoarthritis: Systematic review and meta-analysis of randomized controlled trials. Medicine.

[24] Tonello G., Daglio M., Zaccarelli N., Sottofattori E., Mazzei M., Balbi A. (1996). Characterization and quantitation of the active polynucleotide fraction (PDRN) from human placenta, a tissue repair stimulating agent. J. Pharm. Biomed. Anal..

[25] Chavan A.J., Haley B.E., Volkin D.B., Marfia K.E., Verticelli A.M., Bruner M.W., Draper J.P., Burke C.J., Middaugh C.R. (1994). Interaction of nucleotides with acidic fibroblast growth factor (FGF-1). Biochemestry.

[26] Middlemiss P.J., Gysbers J.W., Rathbone M.P. (1995). Extracellular guanosine and guanosine-5’-trisphoshate increase NGF synthesis and release from cultured mouse neopallial astrocytes. Brain Res..

[27] Irrera N., D’Ascola A., Pallio G., Bitto A., Mannino F., Arcoraci V., Rottura M., Ieni A., Minutoli L., Metro D. (2020). β-Caryophyllene Inhibits Cell Proliferation through a Direct Modulation of CB2 Receptors in Glioblastoma Cells. Cancers.

[28] Thellung S., Florio T., Maragliano A., Cattarini G., Schettini G. (1999). Polydeoxyribonucleotides enhance the proliferation of human skin fibroblasts: Involvement of A_2_ purinergic receptor subtypes. Life Sci..

[29] Muratore O., Schito A.P., Cattarini G., Tonoli E.L., Gianoglio S., Schiappacasse S., Felli L., Picchetta F., Schito G.C. (1997). Evaluation of the trophic effect of human placental polydeoxyribonucleotide on human knee skin fibroblasts in primary culture. Cell Mol. Life Sci..

[30] Gennero L., Denysenko T., Calisti G.F., Vercelli A., Vercelli C.M., Amedeo S., Mioletti S., Parino E., Montanaro M., Melcarne A. (2013). Protective effects of polydeoxyribonucleotides on cartilage degradation in experimental cultures. Cell Biochem. Funct..

[31] Raposio E., Guida C., Coradeghini R., Scanarotti C., Parodi A., Baldelli I., Fiocca R., Santi P.L. (2008). In vitro polydeoxyribonucleotide effects on human pre-adipocytes. Cell Prolif..

[32] Guizzardi S., Galli C., Govoni P., Boratto R., Cattarini G., Martini D., Belletti S., Scandroglio R. (2003). Polydeoxyribonucleotide (PDRN) promotes human osteoblast proliferation: A new proposal for bone tissue repair. Life Sci..

[33] Guizzardi S., Martini D., Bacchelli B., Valdatta L., Thione A., Scamoni S., Uggeri J., Ruggeri A. (2007). Effect of heat deproteinate bone and polynucleotides on bone regeneration: An experimental study on rat. Micron.

[34] Sini P., Denti A., Cattarini G., Daglio M., Tira M.E., Balduini C. (1999). Effect of polydeoxyribonucleotides on human fibroblasts in primary culture. Cell Biochem. Funct..

[35] Belletti S., Uggeri J., Gatti R., Govoni P., Guizzardi S. (2007). Polydeoxyribonucleotide promotes cyclobutane pyrimidine dimer repair in UVB-exposed dermal fibroblasts. Photodermatol. Photoimmunol. Photomed..

[36] Muratore O., Cattarini G., Gianoglio S., Tonoli E.L., Saccà S.C., Ghiglione D., Venzano D., Ciurlo C., Lantieri P.B., Schito G.C. (2003). A human placental polydeoxyribonucleotide (PDRN) may promote the growth of human corneal fibroblasts and iris pigment epithelial cells in primary culture. New Microbiol..

[37] Galeano M., Bitto A., Altavilla D., Minutoli L., Polito F., Calò M., Lo Cascio P., d’Alcontres F.S., Squadrito F. (2008). Polydeoxyribonucleotide stimulates angiogenesis and wound healing in the genetically diabetic mouse. Wound Repair Regen..

[38] Kwon T.R., Han S.W., Kim J.H., Lee B.C., Kim J.M., Hong J.Y., Kim B.J. (2019). Polydeoxyribonucleotides Improve Diabetic Wound Healing in Mouse Animal Model for Experimental Validation. Ann. Dermatol..

[39] Altavilla D., Squadrito F., Polito F., Irrera N., Calò M., Lo Cascio P., Galeano M., La Cava L., Minutoli L., Marini H. (2011). Activation of adenosine A2A receptors restores the altered cell-cycle machinery during impaired wound healing in genetically diabetic mice. Surgery.

[40] Jeong W., Yang C.E., Roh T.S., Kim J.H., Lee J.H., Lee W.J. (2017). Scar Prevention and Enhanced Wound Healing Induced by Polydeoxyribonucleotide in a Rat Incisional Wound-Healing Model. Int. J. Mol. Sci..

[41] Shin D.Y., Park J.U., Choi M.H., Kim S., Kim H.E., Jeong S.H. (2020). Polydeoxyribonucleotide-delivering therapeutic hydrogel for diabetic wound healing. Sci. Rep..

[42] Bitto A., Galeano M., Squadrito F., Minutoli L., Polito F., Dye J.F., Clayton E.A., Calò M., Venuti F.S., Vaccaro M. (2008). Polydeoxyribonucleotide improves angiogenesis and wound healing in experimental thermal injury. Crit. Care Med..

[43] Polito F., Bitto A., Galeano M., Irrera N., Marini H., Calò M., Squadrito F., Altavilla D. (2012). Polydeoxyribonucleotide restores blood flow in an experimental model of ischemic skin flaps. J. Vasc. Surg..

[44] Chung K.I., Kim H.K., Kim W.S., Bae T.H. (2013). The effects of polydeoxyribonucleotide on the survival of random pattern skin flaps in rats. Arch. Plast. Surg..

[45] Lee D.W., Hong H.J., Roh H., Lee W.J. (2015). The Effect of Polydeoxyribonucleotide on Ischemic Rat Skin Flap Survival. Ann. Plast. Surg..

[46] Rubegni P., De Aloe G., Mazzatenta C., Cattarini L., Fimiani M. (2001). Clinical evaluation of the trophic effect of polydeoxyribonucleotide (PDRN) in patients undergoing skin explants. A Pilot Study. Curr. Med. Res. Opin..

[47] Valdatta L., Thione A., Mortarino C., Buoro M., Tuinder S. (2004). Evaluation of the efficacy of polydeoxyribonucleotides in the healing process of autologous skin graft donor sites: A pilot study. Curr. Med. Res. Opin..

[48] Squadrito F., Bitto A., Altavilla D., Arcoraci V., De Caridi G., De Feo M.E., Corrao S., Pallio G., Sterrantino C., Minutoli L. (2014). The effect of PDRN, An adenosine receptor A_2A_ agonist, On the healing of chronic diabetic foot ulcers: Results of a clinical trial. J. Clin. Endocrinol. Metab..

[49] Kim S., Kim J., Choi J., Jeong W., Kwon S. (2017). Polydeoxyribonucleotide Improves Peripheral Tissue Oxygenation and Accelerates Angiogenesis in Diabetic Foot Ulcers. Arch. Plast. Surg..

[50] Kim J.Y., Pak C.S., Park J.H., Jeong J.H., Heo C.Y. (2014). Effects of polydeoxyribonucleotide in the treatment of pressure ulcers. J. Korean Med. Sci..

[51] Haskó G., Linden J., Cronstein B., Pacher P. (2008). Adenosine receptors: Therapeutic aspects for inflammatory and immune diseases. Nat. Rev. Drug Discov..

[52] Burnstock G., Knight G.E., Greig A.V. (2012). Purinergic signaling in healthy and diseased skin. J. Investig. Dermatol..

[53] Ernens I., Léonard F., Vausort M., Rolland-Turner M., Devaux Y., Wagner D.R. (2010). Adenosine up-regulates vascular endothelial growth factor in human macrophages. Biochem. Biophys. Res. Commun..

[54] Ahmed A.H., Jacobson K.A., Kim J., Heppel L.A. (1995). Presence of both A_1_ and A_2a_ adenosine receptors in human cells and their interaction. Biochem. Biophys. Res. Commun..

[55] Olanrewaju H.A., Qin W., Feoktistov I., Scemama J.L., Mustafa S.J. (2000). Adenosine A_2A_ and A_2B_ receptors in cultured human and porcine coronary artery endothelial cells. Am. J. Physiol. Heart Circ. Physiol..

[56] Valls M.D., Cronstein B.N., Montesinos M.C. (2009). Adenosine receptor agonists for promotion of dermal wound healing. Biochem. Pharm..

[57] Altavilla D., Bitto A., Polito F., Marini H., Minutoli L., Di Stefano V., Irrera N., Cattarini G., Squadrito F. (2009). Polydeoxyribonucleotide (PDRN): A safe approach to induce therapeutic angiogenesis in peripheral artery occlusive disease and in diabetic foot ulcers. Cardiovasc. Hematol. Agents Med. Chem..

